# Correlations of Human Epithelial Growth Factor Receptor 2 Overexpression with MUC2, MUC5AC, MUC6, p53, and Clinicopathological Characteristics in Gastric Cancer Patients with Curative Resection

**DOI:** 10.1155/2015/946359

**Published:** 2015-04-28

**Authors:** Kwang Kuk Park, Song I Yang, Kyung Won Seo, Ki Young Yoon, Sang Ho Lee, Hee Kyung Jang, Yeon Myeong Shin

**Affiliations:** ^1^Department of Surgery, Kosin University College of Medicine, 34 Amnam-dong, Seo-gu, Busan 602-703, Republic of Korea; ^2^Department of Pathology, Kosin University College of Medicine, 34 Amnam-dong, Seo-gu, Busan 602-703, Republic of Korea

## Abstract

*Background*. The purpose of this study was to evaluate the relationships between HER2 overexpression in the tumor and MUC2, MUC5AC, MUC6, and p53 status and clinicopathological characteristics of gastric cancer patients. *Methods*. This retrospective study included 282 consecutive patients with gastric cancer who underwent surgery at the Kosin University Gospel Hospital between April 2011 and December 2012. All tumor samples were examined for HER2 expression by immunohistochemistry (IHC) and MUC2, MUC5AC, MUC6, and p53 expression by staining. A retrospective review of the medical records was conducted to determine the correlation between the presence of HER2 overexpression and clinicopathological factors. *Results*. The HER2-positive rate was 18.1%. Although no association was found between HER2 expression and MUC5AC, the expression of MUC2, MUC6, and p53 was significantly correlated with HER2 positivity, respectively (*P* = 0.004, 0.037, 0.002). Multivariate analysis revealed that HER2 overexpression and nodal status were independent prognostic factors. *Conclusions*. HER2 overexpression in gastric carcinoma is an independent poor prognostic factor.

## 1. Introduction

Gastric cancer is still one of the most common and aggressive carcinomas throughout the world with a high mortality, especially in South Korea. At present, an HER2-based concept of tumor biology has been established, and trastuzumab (Herceptin, Genentech/Roche), a monoclonal humanized antibody directed against HER2, is a pivotal agent for the management [1]. In gastric cancer also, many publications have suggested a similar role of HER2 [2–7]. Although many studies have previously evaluated HER2 status in gastric cancer, the patient cohorts and scoring criteria have varied, resulting in discrepancies in HER2 positivity that have ranged from 8.2 to 53.4% [8]. The trastuzumab for gastric cancer (ToGA) trial has assessed HER2-targeting agents for treating advanced gastric cancer [9, 10]. HER2 evaluation becomes an important approach for predicting patient response to HER2-targeting agents. Regarding the clinicopathological features of HER2-positive gastric cancer, HER2 expression and intestinal histological type have shown a high correlation.

When focusing on the cellular origin or differentiation of gastric adenocarcinoma, the expressions of different types of mucins are used as epithelial differentiation markers. Mucins are high molecular weight glycoproteins with oligosaccharides attached to serine or threonine residues of the mucin core protein backbone by O-glycosidic linkages. Mucins can be classified into two categories: transmembrane mucins (MUC1, MUC3, MUC4, MUC12, MUC13, MUC15, MUC16, MUC17, MUC20, and MUC21) and secreted mucins (MUC2, MUC5AC, MUC5B, MUC6, MUC7, and MUC19) [11–13]. The mucins are produced by various epithelial cells and serve protective and lubricating roles. However, in damaged epithelia or tumor cells, there is a loss of polarity that occurs in association with the activation of a proliferation and survival program [14]. Mucin gene expression is relatively organ-specific, and deregulated expression of one or more types of mucins occurs with malignancy [15, 16]. MUC5AC and MUC6 are markers of gastric foveolar cells and antral/cardiac mucous glandular cells, respectively, and they reflect gastric phenotypes. MUC2 exhibit the typical intestinal epithelial cell phenotype, decorating goblet cells and the brush border of intestinal absorptive epithelial cells, [17]. However, the relationship between HER2-positive gastric cancer and mucins was unclear.

The* p53* tumor suppressor gene is the most commonly mutated gene in human tumors [18]. Abnormalities of the* p53* gene have been identified in many malignancies, including gastric carcinomas [19]. The major functions of p53 protein are regulation of the cell cycle and apoptosis and repair of DNA damage. Functional abnormality of p53 is known to be caused by mutation of the* p53 *gene, including loss of heterozygosity (LOH) and DNA methylation, [20] which can affect the biological behavior of the tumor and therefore prognosis.

Although some studies have shown a correlation between HER2 expression and p53 nuclear staining, little is known about the relationship between HER2 positivity and mucins and p53 expression [19, 21]. The purpose of this study was to evaluate the frequency of HER2-positive gastric cancer, by applying the standard scoring criteria in patients with gastric cancer and the relationships between HER2 expression and prognosis, mucins, p53 overexpression, and other clinicopathological features.

## 2. Patients and Methods

From April 2011 to December 2012, a total of 298 consecutive cases with gastric cancer treated by surgical resection without any preoperative therapy were retrieved from the Department of Surgery of Kosin University College of Medicine. The medical records and surgical specimens of these patients were retrospectively evaluated after obtaining approval from the Investigational Review Board of the Kosin University Gospel Hospital. The disease stage was determined according to the (AJCC)-TNM classification (seventh edition) [22]. 3 patients who had gastric cancer recurrence and 13 patients with synchronous multiple primary cancers (e.g., thyroid cancer, colon cancer, and lung cancer) were excluded from the study. The clinicopathological characteristics of each patient were retrieved from hospital information systems, retrospectively. Clinicopathological parameters, including age, gender, tumor location, histological classification, pathological TNM stage, neural invasion, lymphatic invasion, and vascular invasion status, were retrieved from medical charts or pathology reports. Histological classification was determined according to Lauren's classification and the World Health Organization (WHO) classification [23].

### 2.1. Immunohistochemistry (IHC)

Tumor tissues were fixed in 10% formalin and embedded in paraffin. Immunohistochemical staining was carried out using anti-HER-2/neu (Dako, Glostrup, Denmark) as the primary antibody for c-erbB-2. After making slices using a microtome, tissue sections (4 *μ*m) were immersed in xylene solution to remove residual paraffin and hydrated in an alcohol series. Sections were boiled for 5 minutes to retrieve antigenicity in citrate buffer (pH 6.0) and left for 30 minutes at room temperature. After exhausting endogenous peroxidase for 10 minutes with H_2_O_2_ in methyl alcohol, sections were washed thrice with phosphate-buffered saline (PBS). Sections were blocked for 30 minutes with blocking solution (Histostain kit, Zymed Company, San Francisco, CA, USA) at room temperature. Sections were then incubated with anti-HER-2/neu (1 : 200, Dako) at room temperature. After rinsing thrice with PBS, sections were incubated with biotinylated anti-mouse IgG (1 : 300; Zymed). After washing, sections were incubated with avidin-alkaline phosphatase for 7 minutes at 40°C. Sections were visualized with red chromogen at 40°C and counterstained using the Mayer hematoxylin method. Sections were mounted and observed under light microscopy.

### 2.2. Assessment of HER2 Expression

**Figure 1:**
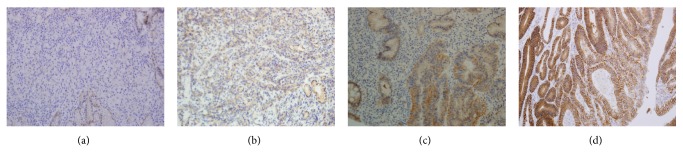
Immunohistochemical analysis of human epidermal growth factor receptor 2 protein expression (a–d). (a) Immunostaining shows no staining on tumor cell membrane. (b) Immunostaining shows positive reaction (1+). (c) Immunostaining shows positive reaction (2+). (d) Immunostaining shows positive reaction (3+) with complete or basolateral membraneous staining.

The amended c-erbB-2 scoring system was applied according to location and degree of completion of staining: 0 points: staining of ≤10% was equivalent; 1 point: incomplete membrane staining of >10%; 2 points: weak-to-moderate complete staining of the membrane; and 3 points: strong complete staining of the membrane. An immunohistochemistry score of 3+ or immunohistochemistry score of 2+ was defined as overexpression of HER2 (Figure 1).

### 2.3. MUC2, MUC5AC, MUC6, and p53 Expression Profile in Gastric Carcinoma

Mucin and p53 IHC staining was performed by the tyramide signal amplification-avidin-biotin complex method [24]. We used monoclonal antibodies against MUC5AC (Novocastra, Newcastle-upon-Tyne, UK; diluted 1 : 100) as a marker for gastric faveolar cells, MUC6 (Novocastra; 1 : 100) as a marker for gastric mucous neck cells and pyloric glands, MUC2 (Novocastra; 1 : 100) as a marker for intestinal goblet cells, and p53 (1 : 100, clone DO-7; Dako). The expressions of MUC2, MUC5AC, and MUC6 were regarded as positive when more than 10% of the area was positively stained [17]. Overexpression of p53 was regarded as positive when more than 10% of tumor cells displayed nuclear immunostaining [25].

### 2.4. Statistical Analysis

The statistical evaluation was performed using Spearman's correlation test to analyze rank date. Kaplan-Meier survival plots were generated and comparisons between survival curves were made with the log-rank statistics. Cox's proportional hazards model was used for the multivariate analysis. SPSS 18.0 software (SPSS Inc., Chicago, IL, USA) was applied to analyse all data and *P* < 0.05 was considered to indicate a statistically significant difference.

## 3. Results

### 3.1. Demographic Characteristics

The studied population consisted of 187 men and 95 women. In the 282 cases enrolled in the study, the median age of the patients at diagnosis was 60 years (range 34–85 years) and all patients were Korean. The demographics and tumor-related factors are summarized in Table 1. Tumor location in the stomach was lower third in 164, middle third in 71, and upper third in 45 cases, two of which were located at the gastroesophageal junction. In accordance with the World Health Organization's classification standards, 167 patients (60.9%) had tubular adenocarcinoma and 106 patients (37.6%) had poorly cohesive carcinoma. According to the pathological depth of tumor, 138 patients (48.9%) were pT1a, 37 (13.1%) were pT1b, 15 (5.3%) were pT2, 55 (19.5%) were pT3, 36 (12.8%) were pT4a, and 1 (0.4%) was pT4b. Regarding the tumor stage, 158 (56.0%) were stage I, 63 (22.3%) stage II, 61 (15.2%) stage III.

### 3.2. HER2 Status and the Results of MUC2, MUC5AC, MUC6, and p53

Immunohistochemical analysis was performed to examine the expression of HER2 in all the cases. The numbers of patients with an HER2 score of 0, 1+, 2+, and 3+ were 207 (73.4%), 24 (8.5%), 5 (1.8%), and 46 (16.3%). The percent of positive stain for HER2 and p53 positive rate was 18.1% (51 of 282) and 71.9% (202/282). The positive rates of MUC2, MUC5AC, and MUC6 were 18.5% (52/282), 71.9% (202/282), and 23.6% (66/282). The expression of HER2, MUC2, MUC5AC, MUC6, and p53 is summarized in Table 2.

### 3.3. Correlation of HER2 Status with Clinicopathological Features

The correlation between HER2 status and the clinicopathological features is shown in Table 1. The frequency of HER2 positivity correlated with WHO classification, Lauren classification, depth of tumor, nodal stage, TNM stage, and lymphatic invasion. According to Lauren's classification, HER2 overexpression was more often detected in the intestinal histological type (24.0%) than in the diffuse type (14.0%), indeterminate type (28.6%), or mixed type (2.6%). HER2 overexpression was more commonly observed in patients with lymph node metastasis (N0 disease, 13.2%; N1 disease, 13.3%; N2 disease, 30.3%; N3 disease, 35.1%; *P* = 0.003). And differences in frequency of HER2 overexpression among the T stages also were estimated to be significant (*P* = 0.036). On TNM stage, HER2 overexpression was more commonly observed in patients with advanced stage (*P* = 0.009). HER2-positive tumors tended to present with lymphatic invasions with statistical significance (*P* = 0.027). The presence of HER2 overexpression in the tumor was not influenced by tumor location or tumor size.

### 3.4. Correlation of HER2 Expression with MUC2, MUC5AC, MUC6, and p53

The results of the analysis of the expression of the three mucin markers based on mucin expression are shown in Table 2. Of the three markers, MUC2 expression and MUC6 expression were significantly correlated with HER2 positivity (*P* = 0.004, *P* = 0.043). There was no correlation between the MUC5AC and HER2 overexpression. The p53 overexpression was significantly correlated with HER2 positivity (*P* = 0.034).

### 3.5. Prognostic Significance of HER2, MUC2, MUC5AC, MUC6, and p53 Expression in Gastric Carcinoma

**Figure 2:**
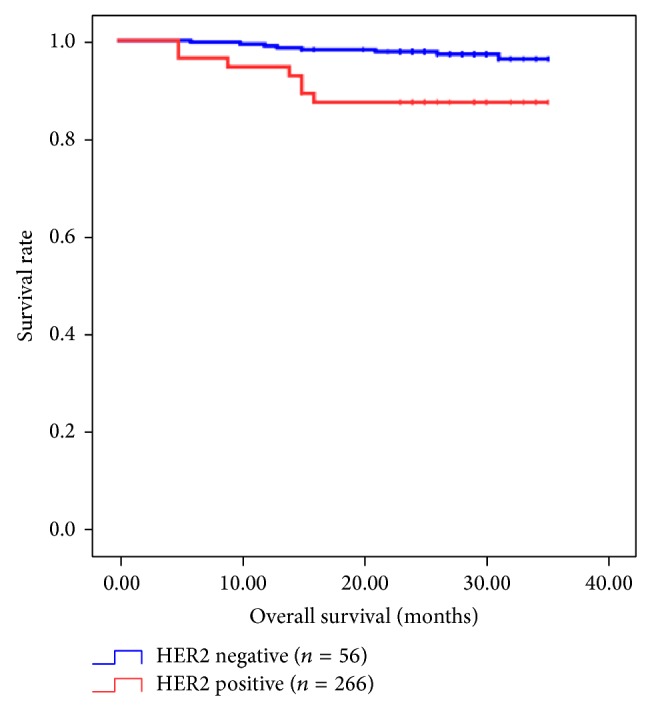
Kaplan-Meier survival curves according to the HER2 overexpression in patients with gastric carcinoma.

Univariate analysis of factors related to overall survival in gastric carcinoma is summarized in Table 3. Patients with HER2 overexpression had significantly worse survival than those without HER2 overexpression (*P* = 0.001) (Figure 2). Other factors significantly correlated with survival were Lauren classification, TNM stage, depth of invasion, nodal status, and lymphatic invasion. There was no significant difference in survival related to MUC2, MUC5AC, MUC6, and p53 expression. The five factors (HER2 overexpression, Lauren classification, depth of invasion, nodal status, and lymphatic invasion) selected from univariate analysis, based on a *P* value < 0.05, were entered into the Cox proportional hazards model. This multivariate analysis showed that HER2 expression (*P* = 0.024) and nodal status (*P* < 0.001) were significant risk factors affecting the outcome (Table 3).

## 4. Discussion

The c-erbB-2 gene is a protooncogene located on chromosome 17. It expresses HER2/*neu *protein, one of the epithelial growth factor receptor (EGFR) families, and has tyrosine kinase (TK) activity, which mediates cancer proliferation [26]. HER2 gene amplification and protein overexpression have been suggested as the targets for a therapy with anti-HER2 humanized monoclonal antibody (trastuzumab) in various cancers; [27–35] in those studies, a very wide range of HER2 expression has been described with controversial data for most cancer types [34]. Recently, in an open-label international phase 3 randomized controlled trial (ToGA trial) undertaken in 122 centers in 24 countries, patients with advanced gastric or gastroesophageal junction carcinomas have been studied in order to verify if their tumors showed overexpression of HER2 protein by immunohistochemistry or gene amplification by fluorescence in situ hybridization (FISH) [9]. In ToGA study, the HER2-positive ratio was higher in tumors at the gastroesophageal junction than in gastric cancer (33.2 versus 20.9%). However, it has been shown that the addition of trastuzumab to chemotherapy improved survival in patients with advanced gastric or gastroesophageal junction carcinomas compared with chemotherapy alone [9, 36–38].

Since the overexpression of HER2 in gastric cancer was first published in 1986 [39], many studies have reported the frequency of HER2 positivity in gastric cancer patients from various regions throughout the world. According to the review by Jorgensen and Hersom [8], the positive rate ranged from 4% to 53% by IHC alone and from 9% to 18% when ISH (in situ hybridization) was included. But according to what was reported by Sheng et al. [40], detected by both IHC and ISH, the HER2-positive rate was 13%. In the present study, the rate of HER2 positivity was estimated to be 18.1%. Of the 51 HER2-positive tumors in the present study, 36 were of intestinal type and 12 were of diffuse type according to Lauren's classification. These data are consistent with previous reports that the intestinal type showed a higher rate of HER2 positivity than the diffuse/mixed type [37, 41–47]. A strong correlation between HER2 positivity and intestinal histological type is also supported by our finding. Furthermore, HER2 overexpression was related to depth of invasion of tumor, lymph node metastasis, pTNM stage, and lymphatic invasion presenting aggressive features of the tumor (Table 2). Lymph node metastasis was significantly more frequent in patients with poorly differentiated tumor histology. Several previous studies have reported the existence of a relationship between the presence of lymph node metastasis and tumor HER2 overexpression [42, 48]. This finding suggests that tumor biology in the subgroup of patients with tumor HER2 overexpression predisposes to spread via the lymphatic system. This finding suggests the potential clinical benefit of HER2-targeted therapy in the adjuvant or neoadjuvant setting for patients with node-positive gastric cancer.

In breast cancer, overexpression of the HER2 gene was associated with poor outcomes, higher mortality, and increased higher recurrence and metastasis [1, 49, 50]. It has been generally reported that HER2 overexpression is correlated with aggressive biological behavior and poor prognosis, despite some contradictory results in gastric carcinoma [2, 5, 45, 51–60]. Our study confirmed that the HER2 positivity was associated with lymphatic invasion and lymph node metastases (including pN stage) in agreement with previous studies [53, 54, 56, 59, 61]. Our findings suggest that HER2 overexpression is associated with a progression of gastric cancer.

However, the association between HER2 status and prognosis in gastric cancer remains controversial, and a correlation between HER2 amplification or overexpression and favorable survival has only been shown in a few studies [9, 62, 63]. Some studies have indicated that HER2 overexpression is strongly associated with differentiated or intestinal type gastric cancers, which generally have a better prognosis than undifferentiated or diffuse type cancers. This may be the major source of controversies surrounding the prognostic value of HER2 overexpression. But most published studies assessing this association have shown a poor prognosis in HER2-positive gastric cancers. In our study, HER2 overexpression was correlated with poor prognosis.

The present study has examined the association between the mucin markers and HER2 status of gastric cancer. Of the three mucin markers that we examined, the expression of MUC2 (goblet cells) and MUC6 (mucous neck cells, pyloric glands) was significantly correlated with HER2 positivity.

Because MUC2 was strongly correlated with the intestinal histological type in our study (*P* = 0.004), it seems that the correlation between MUC2 and HER2 expression may reflect a linkage between the intestinal differentiation of cancer cells and HER2 expression.

The* p53* gene is also located on chromosome 17 like the c-erbB-2 gene. It is a representative tumor suppressor gene, and mutations of this gene are found out in most tumors originating from the gastrointestinal system, urogenital system, and skin [64]. The wild-type* p53 *gene is involved in the differentiation, proliferation, and apoptosis of cells, whereas the mutant type is considered to be the cause of atypical cell growth [65]. Accumulation of p53 protein in the nuclei of carcinoma cells is known to correlate well with the presence of mutations in the* p53* gene [36, 38]. Our study demonstrated a strong correlation between p53 overexpression and HER2 positivity, suggesting a possible role of p53 abnormality in the development of HER2-positive gastric cancer. These findings are consistent with previous studies, which have reported a correlation between p53 nuclear staining and HER2 positivity [19, 21]. Intriguingly, some studies also reported a linkage between alterations of p53 and the intestinal histological type. Consistently, our study confirmed that p53 overexpression was more often found in the intestinal type of gastric cancer [25, 53]. These results suggest that the intestinal differentiation of cancer cells, as well as HER2 and MUC2 expression, may also be linked to the expression of p53. The significance of the expression of these molecules in regard to tumor biology and prognosis needs to be determined by further studies.

This study had some limitations. Even though we focused on gastric cancer treated by curative resection, we could not evaluate the effect of adjuvant chemotherapy. Most early stage gastric cancer patients were not treated with adjuvant chemotherapy, and patients with more advanced stages were treated with heterogeneous adjuvant chemotherapy protocol. The influence of tumor location was not analyzed due to the small number of cardiac origin patients (0.6%) included in this study There is a relatively low incidence of proximal gastric cancer in Asian patients compared to the incidence in Western countries. Therefore, there were too few patients to evaluate differences in tumor location. And this study includes the relatively short follow-up time, particularly for maintenance of oncologic issues. A long term follow-up is needed to determine the association between HER2 expression and the mucin markers and p53 of gastric cancer prognosis.

## 5. Conclusion

In summary, we assessed the HER2 status in 282 samples from consecutive surgical cases of gastric cancer. The total HER2-positive rate was 18.1%. HER2 overexpression was correlated with Lauren classification, TNM stage, depth of invasion, nodal status, and lymphatic invasion. And MUC2 and MUC6 were significantly correlated with HER2 positivity. HER2 status was found to be associated with several clinicopathological characteristics related to the invasive behavior of gastric cancer, especially lymph node metastasis and lymphatic invasion. This finding suggests the potential clinical benefit of HER2-targeted therapy in the adjuvant or neoadjuvant setting for patients with node-positive gastric cancer.

And this study shows that HER2 overexpression is an independent poor prognostic factor in gastric carcinoma. Therefore, HER2 expression may be a useful marker to predict the outcome of patients with surgically resected gastric carcinoma.

## Figures and Tables

**Table 1:** Correlation between HER2 overexpression and clinicopathologic parameters of gastric carcinoma.

Variables	*N* (%)	HER2 positive		*P* value
		Positive (%) (*n* = 56)	Negative (%) (*n* = 266)	
Age^a^	60.51 ± 10.86	59.70 ± 9.85	60.68 ± 11.07	0.540
Gender				0.546
Male	187 (66.3)	34 (18.2%)	153 (81.8%)	
Female	95 (33.7)	17 (17.9%)	78 (82.1%)	
Location				0.173
Upper	45 (16.0)	12 (26.7%)	33 (73.7%)	
Middle	71 (25.2)	8 (11.3%)	63 (88.7%)	
Lower	164 (58.2)	31 (18.9%)	133 (81.1%)	
EGJ^b^	4 (1.2)	1 (25.0%)	3 (75.0%)	
WHO classification				**0.022**
Tubular	167 (60.9)	32 (19.2%)	135 (80.8%)	
Mucinous	1 (0.4)	0 (0.0%)	1 (100.0%)	
Poorly cohesive	106 (37.6)	15 (14.2%)	91 (85.8%)	
Papillary	6 (2.1)	2 (33.3%)	4 (66.7%)	
Lauren classification				**0.010**
Intestinal	150 (53.2)	36 (24.0%)	114 (76.0%)	
Diffuse	86 (30.5)	12 (14.0%)	74 (86.0%)	
Mixed	39 (13.8)	1 (2.6%)	38 (97.4%)	
Indeterminate	7 (2.5)	2 (28.6%)	5 (71.4%)	
Size^a^	3.48 ± 3.06	3.87 ± 3.15	3.40 ± 3.04	0.293
Tumor size				0.176
5 cm <	61 (21.6)	14 (23.0%)	47 (77.0%)	
5 cm ≥	221 (78.4)	37 (16.7%)	184 (83.3%)	
Depth of tumor (T stage)				**0.036**
T1a	138 (48.9)	20 (14.5%)	118 (85.5%)	
T1b	37 (13.1)	9 (24.3%)	28 (75.7%)	
T2	15 (5.3)	1 (6.7%)	14 (93.3%)	
T3	55 (19.5)	9 (16.4%)	46 (83.6%)	
T4a	36 (12.8)	11 (30.6%)	25 (69.4%)	
T4b	1 (0.4)	1 (100.0)	0 (0.0)	
Nodal stage (N)				**0.003**
N0	182 (64.5)	24 (13.2%)	158 (86.8%)	
N1	30 (10.6)	4 (13.3%)	26 (86.7%)	
N2	33 (11.7)	10 (30.3%)	23 (69.7%)	
N3	37 (13.1)	13 (35.1%)	24 (64.9%)	
Stage (TNM)				**0.027**
I	158 (56.0)	22 (13.9%)	136 (86.1%)	
II	63 (22.3)	11 (17.5%)	52 (82.5%)	
III	61 (21.7)	18 (29.5%)	43 (70.5%)	
Neural invasion				0.345
Yes	56 (21.0)	8 (14.3%)	48 (85.7%)	
No	211 (79.0)	43 (20.4%)	168 (79.6%)	
Lymphatic invasion				**0.011**
Yes	83 (31.1)	24 (28.9%)	59 (71.1%)	
No	184 (68.9)	27 (14.7%)	157 (85.3%)	
Vascular invasion				0.105
Yes	14 (5.2)	5 (35.7%)	9 (64.3%)	
No	253 (94.8)	46 (18.2%)	207 (81.8%)	
Recurrence				**0.005**
Yes	31 (11.0)	12 (38.7%)	19 (61.3%)	
No	251 (89.0)	39 (15.5%)	212 (84.5%)	

^a^Age and size were reported as the mean ± SD.

^b^Esophagogastric junction.

**Table 2:** Correlation of HER2 overexpression with MUC2, MUC5AC, MUC6, and p53.

Variables	*N* (%)	HER2 positive		*P* value
		Positive (%) (*n* = 51)	Negative (%) (*n* = 231)	
p53				** 0.034**
Positive	210 (74.5%)	44 (21.0%)	166 (79.0%)	
Negative	72 (25.5%)	7 (9.7%)	65 (90.3%)	
MUC2				**0.004**
Positive	52 (18.5%)	17 (32.7%)	35 (67.3%)	
Negative	229 (81.5%)	33 (14.4%)	196 (85.6%)	
MUC5AC				0.731
Positive	202 (71.9%)	39 (17.4%)	167 (82.7%)	
Negative	79 (28.1%)	16 (16.7%)	64 (81.0%)	
MUC6				**0.043**
Positive	64 (22.9%)	17 (26.6%)	47 (73.4%)	
Negative	216 (77.1%)	33 (15.3%)	183 (84.7%)	

**Table 3:** Univariate and multivariate analysis of clinicopathological variables for the survival of the patients with gastric carcinoma.

Variables	Relative risk (95% CI)	*P* value
Univariate		
TNM stage	5.317 (2.936–9.630)	*P* < 0.001
Tumor size (5 cm <)	2.962 (1.051–8.345)	*P* = 0.040
Depth of invasion (T3-T4)	2.453 (1.649–3.647)	*P* < 0.001
Nodal status (N stage)	3.973 (2.375–6.647)	*P* < 0.001
p53 expression (+)	0.287 (0.080–1.024)	*P* = 0.054
MUC2 expression (+)	1.786 (0.565–5.640)	*P* = 0.323
MUC5AC expression (+)	1.728 (0.488–6.125)	*P* = 0.397
MUC6 expression (+)	1.257 (0.397–3.977)	*P* = 0.697
HER2 overexpression (+)	4.673 (1.690–12.922)	*P* = 0.003
Lauren classification	1.914 (1.099–3.337)	*P* = 0.022
Vascular invasion (+)	12.218 (4.143–36.029)	*P* < 0.001
Lymphatic invasion (+)	17.917 (4.033–79.610)	*P* < 0.001
Neural invasion (+)	5.049 (1.830–13.926)	*P* = 0.002

Multivariate: Cox regressional hazard model		
HER2 overexpression (+)	3.394 (1.173–9.819)	*P* = 0.024
Nodal status (N stage)	3.840 (2.278–6.473)	*P* < 0.001
